# Operation time and clinical outcomes for open infrarenal abdominal aortic aneurysms to remain stable in the endovascular era

**DOI:** 10.3389/fcvm.2023.1213401

**Published:** 2023-11-14

**Authors:** M. Gruber, A. Sotir, J. Klopf, S. Lakowitsch, C. Domenig, A. Wanhainen, C. Neumayer, A. Busch, W. Eilenberg

**Affiliations:** ^1^ Division of Vascular Surgery, Department of General Surgery, Medical University of Vienna, Vienna, Austria; ^2^ Department of General, Visceral, Transplant, Vascular, and Pediatric Surgery, University Hospital Würzburg, Würzburg, Germany; ^3^ Department of Surgical Sciences, Vascular Surgery, Uppsala University, Uppsala, Sweden; ^4^ Department of Surgical and Perioperative Sciences, Surgery, Umeå University, Umeå, Sweden; ^5^ Department of Visceral, Thoracic and Vascular Surgery, Medical Faculty Carl Gustav Carus and University Hospital, Technical University Dresden, Dresden, Germany

**Keywords:** abdominal aortic aneurysm, open repair, endovascular repair, surgical education, outcomes of aortic surgery

## Abstract

**Objective:**

Endovascular aortic repair (EVAR) has become a routine procedure worldwide. Ultimately, the increasing number of EVAR cases entails changing conditions for open surgical repair (OSR) regarding patient selection, complexity, and surgical volume. This study aimed to assess the time trends of open abdominal aortic aneurysm (AAA) repair in a high-volume single center in Austria over a period of 20 years, focusing on the operation time and clinical outcomes.

**Materials and methods:**

A retrospective analysis of all patients treated for infrarenal AAAs with OSR or EVAR between January 2000 and December 2019 was performed. Infrarenal AAA was defined as the presence of a >10-mm aortic neck. Cases with ruptured or juxtarenal AAAs were excluded from the analysis. Two cohorts of patients treated with OSR at different time periods, namely, 2000–2009 and 2010–2019, were assessed regarding demographical and procedure details and clinical outcomes. The time periods were defined based on the increasing single-center trend toward the EVAR approach from 2010 onward.

**Results:**

A total of 743 OSR and 766 EVAR procedures were performed. Of OSR cases, 589 were infrarenal AAAs. Over time, the EVAR to OSR ratio was stable at around 50:50 (*p* = 0.488). After 2010, history of coronary arterial bypass (13.4% vs. 7.2%, *p* = 0.027), coronary artery disease (38.1% vs. 25.1%, *p* = 0.004), peripheral vascular disease (35.1% vs. 21.3%, *p* = 0.001), and smoking (61.6% vs. 34.3%, *p* < 0.001) decreased significantly. Age decreased from 68 to 66 years (*p* = 0.023). The operation time for OSR remained stable (215 vs. 225 min, first vs. second time period, respectively, *p* = 0.354). The intraoperative (5.8% vs. 7.2%, *p* = 0.502) and postoperative (18.3% vs. 20.8%, *p* = 0.479) complication rates also remained stable. The 30-day mortality rate did not change over both time periods (3.0% vs. 2.4%, *p* = 0.666).

**Conclusion:**

Balanced EVAR to OSR ratio, similar complexity of cases, and volume over the two decades in OSR showed stable OSR time without compromise in clinical outcomes.

## Introduction

The modern era of vascular surgery has been designated with a robust implementation of endovascular aortic repair (EVAR) procedures, which have nowadays become a favorable treatment solution for both elective and ruptured abdominal aortic aneurysms (AAAs) in the majority of centers (1).

Several randomized and observational trials were designed to compare the early and late outcomes of the patients undergoing either open surgical repair (OSR) or EVAR (1, 2). Following this, a benefit of EVAR vs. OSR with regard to significantly reduced 30-day mortality rates, length of hospital stay, and complication rates could be confirmed (3). However, in a long-term perspective, the survival benefit following EVAR vs. OSR could not be sustained after the first postoperative year, additionally underlying a potential drawback of EVAR, which has shown to be associated with higher reintervention rates during the 6-year follow-up period (4, 5). The achievement of a satisfactory long-term outcome following EVAR has required substantial modernization in stent-graft technology and has additionally been supported by an extending learning curve (1).

As attributable to all surgical specialties, the introduction and robust implication of modern surgical approaches can lead to a decreased use of the previously used standard-of-care techniques. Considering this, the extensive use of EVAR in routine clinical practice may lead to practitioners having less open surgical training and experience, which could lead to a prolonged OSR time (OSRT) and hospitalization time and a worse clinical outcome following OSR (2, 6–8).

Therefore, this study aimed to analyze the time trends of open AAA repair in a high-volume single center in Austria over a 20-year time period, focusing on operation times and clinical outcomes.

## Materials and methods

### Consecutive patient cohort

A retrospective single-center analysis of all consecutive patients treated for AAAs at a single institution between January 2000 and December 2019 was performed. The exclusion criteria were juxtarenal or ruptured AAAs. First, all cases matching the inclusion criteria—infrarenal AAAs treated with EVAR or OSR—were identified. An infrarenal AAA was defined by the presence of a >10-mm aortic neck and cases involving infrarenal aortic clamping. Treatment decision was made based on anatomic suitability, the general health condition of the patient, the consensus of the institutional interdisciplinary vascular board, and, in some cases, the surgeon’s and patient’s preference. OSR was performed by one of the five senior surgeons during the observational period, and has also included teachning cases. The proportion of residents in training for vascular surgery to senior surgeons remained stable, with a ratio of 2:5 over time.

After identifying all EVAR and OSR cases matching the inclusion criteria, the cohort of patients treated with OSR was selected and subjected to a more detailed analysis of study outcome measures. This study was approved by the Ethics Committee of the Medical University of Vienna, Austria, and was performed in accordance with the principles of the Declaration of Helsinki and STROBE guidelines (9).

### Study outcomes

Demographic data, including comorbidities, were retrieved from medical records. The anatomical characteristics of the aneurysm (maximum aneurysm diameter, aortic neck length, stenosis of the inferior mesenteric artery (IMA), and presence of common iliac artery aneurysm) were recorded using the preoperative computed tomography images. Morphological characteristics were used to define inflammatory aortic aneurysms using computed tomography angiography images, including the presence of “the mantle sign,” a thickened wall from chronic inflammatory cells, and dense peri-aneurysmal fibrosis extending to the posterior wall. The procedure details, including operation time, intraoperative complications, types of grafts, hostile abdomen, and clamp location, were extracted from the operative reports. Intraoperative complications were defined as the incidence of bleeding, anastomotic leakage, organ lesion, cardiovascular event, distal anastomotic occlusion (DAO), or intestinal ischemia. A cardiovascular event was defined as the incidence of intraoperative myocardial infarction, stroke, or cardiac arrest. Postoperative complications and 30-day mortality rates were assessed using clinical discharge letters and a medical record system. Postoperative complications were defined as any complication that scored ≥3 a in accordance with the Clavien–Dindo score (2). The outcome measures of a previously outlined study were compared for the OSR cohort at two time periods, namely, 2000–2009 and 2010–2019. Time periods were defined based on the increasing single-center trend toward the EVAR approach from 2010 onward.

### Statistical analysis

The Kolmogorov–Smirnov test was used to assess the normality of data distribution, with all continuous variables showing abnormal distribution. The variables were presented as medians and interquartile ranges and compared between two time periods using the Mann–Whitney *U*-test. For the categorical variables, absolute numbers and percentages were reported and analyzed using the *χ*^2^ test. Univariate and multivariate linear regression models were used to analyze the impact of assessed variables on operation time. Statistical significance was set at a *p*-value of <0.05. SPSS software version 27 (IBM, New York, NY, USA) was used for statistical analysis.

## Results

A total of 1,509 patients treated for AAAs between January 2000 and December 2019 were identified. Of these, 743 (49.2%) patients had undergone OSR, and 766 (50.8%) patients had undergone EVAR (*p *= 0.488). Among the cohort of patients that had undergone OSR, 598 patients (81%) had had infrarenal AAAs. A total of 145 patients treated for juxtarenal AAAs were excluded from the analysis. A juxtarenal AAA was defined as an aneurysm with an infrarenal neck <10 mm.

Therefore, 598 (81%) patients that had undergone OSR for infrarenal AAAs constituted the study population of interest. This was further classified into two groups according to the time period in which OSR was performed. The patients operated on between the years 2000 and 2009 were classified into time period 1 (TP1), and those that had undergone repair between the years 2010 and 2019 were classified into time period 2 (TP2).

### Demographic details

The median age at the time of surgery increased significantly from 66 to 68 years when comparing TP1 and TP2, respectively (*p *= 0.023). In addition, the proportion of male patients decreased (92.7% vs. 83.6%, *p *= 0.001). Table 1 depicts the demographical details and comorbidities.

**Table 1:** Demographic details of patients and AAA morphological characteristics.

	OSR (infrarenal AAA) TP1 (2000–2009) *N* = 328, 53 missing	OSR (infrarenal AAA) TP2 (2010–2019) *N* = 207, 15 missing	*p*-value
Demographics			
Age (years), median	**66 (61–71)**	**68 (62–73)**	**0.023**
Male (%)	**304 (92.7)**	**173 (83.6)**	**0.001**
Body mass index (kg/m^2^)	27 (25–30)	27 (25–30)	0.652
Comorbidities			
Arrhythmia	35 (10.7)	24 (11.6)	0.740
CAVD	64 (19.5)	48 (23.2)	0.309
CABG	**44 (13.4)**	**15 (7.2)**	**0.027**
Diabetes	43 (13.1)	34 (16.4)	0.287
Dyslipidemia	**162 (49.4)**	**133 (64.3)**	**0.002**
Renal disease	29 (8.8)	26 (12.6)	0.168
Dialysis	7 (2.1)	3 (1.4)	0.569
Hypertension	251 (76.5)	167 (80.7)	0.258
CAD	**125 (38.1)**	**52 (25.1)**	**0.004**
Cancer	30 (9.1)	19 (9.2)	0.990
Neurologic disease	26 (7.9)	20 (9.7)	0.464
Smoker	**202 (61.6)**	**71 (34.3)**	**<0.001**
Pulmonary disease	80 (24.4)	58 (28.0)	0.350
PAD	**115 (35.1)**	**44 (21.3)**	**0.001**
AAA morphology			
Neck length (mm)	**21 (11–30)**	**28 (20–40)**	**<0.001**
IMA stenosis	0	6 (2.9)	0.825
+Iliac aneurysm	100 (30.5)	53 (25.6)	0.223
Diameter (mm)	57 (52–65)	56 (52–65)	0.290
+AIOD	**113 (34.5)**	**34 (16.4)**	**<0.001**
RetroLRV	8 (2.4)	6 (2.9)	0.746
Inflammatory AAA	**40 (12.2)**	**14 (6.8)**	**0.042**
Symptomatic AAA	**81 (24.7%)**	**35 (16.9%)**	**0.010**

CAVD, cerebral arterial vascular disease; RetroLRV, retroaortal left renal vein.

Data are presented as *n* (%) or median + interquartile range unless stated otherwise; *p*-values are obtained by the chi-squared test and Mann–Whitney *U*-test (*).

Bold values represent the variables which were significantly different between 2 time-points.

### Aortic aneurysm morphology

Significant differences between TP1 and TP2 were found in the neck length (21 vs. 28 mm, respectively, *p *< 0.001), the proportion of patients presenting aortoiliac occlusive disease (AIOD) (34.5% vs. 16.4%, *p *< 0.001), and the percentage of inflammatory AAAs (12.2% vs. 6.8%, *p *= 0.042, Table 1).

### Procedure details and OSRT

**Figure 1:**
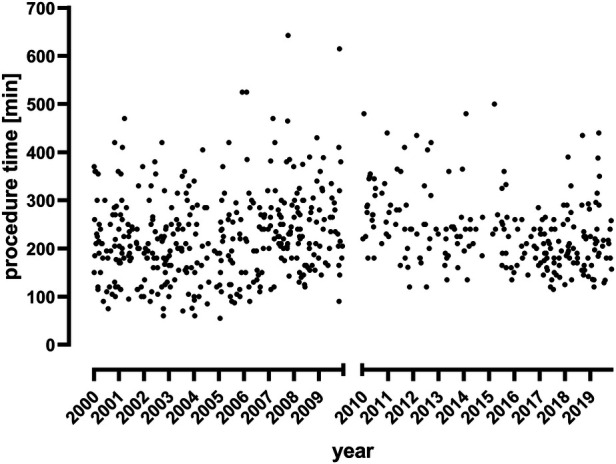
Procedure times in infrarenal AAAs.

**Figure 2:**
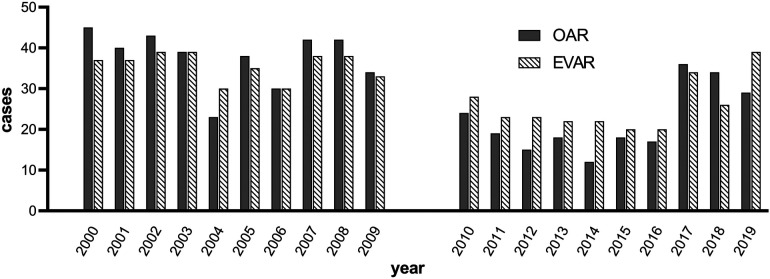
OSR and EVAR cases over time.

The overall OSRT was stable during the observation period (215 min for TP1 vs. 225 min for TP2, *p *= 0.354, Figure 1 and Table 2). In a subgroup analysis, OSRT was lower for bifurcated grafts (249 vs. 230 min, *p* = 0.008), whereas it remained stable for tube grafts (185 vs. 190 min., *p *= 0.059). At the same time, the proportion of tube grafts vs. bifurcated grafts decreased significantly from 51.5% at TP1 to 24.6% at TP2 (*p *≤ 0.001). The transabdominal approach was favored during the study period and increased significantly at TP2 (81.1% vs. 99.5%, *p *< 0.001). OSRT remained stable in patients with a transabdominal approach (195 vs. 200 min, *p *= 0.919). Comparing the number of performed surgeries in both decades, a significant reduction of surgical cases in the second decade (from 2010 to 2019) was found. This is true for both surgical approaches, OSR (*p *< 0.001) and EVAR (*p *= 0.003) (Figure 2). The median clamp time increased by 5 min (55 vs. 60 min, *p *= 0.001). Reconstruction of the IMA was performed significantly more often in the second decade (3.7% vs. 17.4%, *p *< 0.001). No change to OSRT could be found in this subgroup (255 vs. 240 min, *p *= 0.596).

**Table 2:** Procedure details and clinical outcomes of patients.

	OSR (infrarenal AAA) TP1 (2000–2009) *N* = 328, 53 missing	OSR (infrarenal AAA) TP2 (2010–2019) *N* = 207, 15 missing	*p*-value
Procedure details			
Procedure time (min)	215 (165–284)	225 (180–265)	0.354
Procedure time tube graft (min)	185 (123–236)	190 (165–240)	0.059
Procedure time for Y-graft (min)	**249 (200–315)**	**230 (185–270)**	**0.008**
Tube graft	**169 (51.5)**	**51 (24.6)**	**<0.001**
Y-graft	**159 (48.5)**	**155 (74.9)**	** **
Elective	314 (95.7)	202 (97.6)	0.330
Hostile abdomen	87 (26.5)	61 (29.5)	0.458
Clamp time (min)	**55 (35–65)**	**60 (45–80)**	**0.001**
RVR	58 (17.7)	30 (14.5)	0.332
Reconstruction RV	5 (1.5)	1 (0.5)	0.272
Reconstruction IMA	**12 (3.7)**	**36 (17.4)**	**<0.001**
Reconstruction RAS	5 (1.5)	5 (2.4)	0.444
Transabdominal approach	**266 (81.1)**	**206 (99.5)**	**<0.001**
Retroperitoneal approach	**62 (18.9)**	**1 (0.5)**	** **
Clinical outcome			
Hospitality (days)	**10 (8–12)**	**11 (9–14)**	**<0.001**
Intraoperative complications	19 (5.8)	15 (7.2)	0.502
Postoperative complications	60 (18.3)	43 (20.8)	0.479
ICU stay (days)	**1 (1–2)**	**2 (1–3)**	**0.001**
Postoperative dialysis	13 (4.0)	5 (2.4)	0.333
Postoperative cardiac event	13 (4.0)	3 (1.4)	0.096
30-day mortality	10 (3.0)	5 (2.4)	0.666

IQR, interquartile range; Y-graft, bifurcational prosthesis; RVR, renal vein resection; RV, renal vein; RAS, renal arterial stenosis; FU, follow-up.

Data are presented as *n* (%) or median + interquartile range unless stated otherwise; *p*-values are obtained by the chi-squared test and Mann–Whitney *U*-test (*).

Bold values represent the variables which were significantly different between 2 time-points.

### Perioperative and postoperative outcomes

Hospitalization stay increased by 1 day to a median of 11 days in TP2 (*p *< 0.001, Table 2). In addition, postoperative intensive care unit (ICU) stay increased by 1 day in TP2 (*p *= 0.001). The intraoperative complication rate remained stable in TP1 and TP2 (5.8% vs. 7.2%, respectively, *p *= 0.502). Table 3 gives a detailed overview of intraoperative complications, which showed no significant difference between TP1 and TP2. The incidence rate of postoperative complications did not change (18.3% vs. 20.8%, *p *= 0.479). No changes regarding 30-day mortality rates could be found within the observation period (3.0% vs. 2.4%, *p *= 0.666).

**Table 3:** Intraoperative complications.

	OSR (infrarenal AAA) TP1 (2000–2009) *N* = 376	OSR (infrarenal AAA) TP2 (2010–2019) *N* = 222	*p*-value
Bleeding	9 (2.4)	5 (2.3)	0.912
Anastomotic leakage	8 (2.1)	8 (3.6)	0.280
Organ lesion	5 (1.3)	4 (1.8)	0.647
Cardiovascular event	1 (0.3)	2 (0.9)	0.288
DAC	3 (0.8)	4 (1.8)	0.270
Intestinal ischemia, *n* (%)	1 (0.3)	0 (0.0)	0.442

Data are presented as *n* (%) or median +IQR unless stated otherwise; *p*-values are obtained by the chi-squared test.

DAC, distal anastomotic occlusion.

### Univariate and multivariate linear regression analyses for OSRT

It was found from the univariate analysis that OSRT was associated with hostile abdomen (*p *= 0.061), inflammatory AAA (*p *= 0.002), iliac peripheral arterial disease (PAD) (*p *< 0.001), iliac aneurysm (*p *< 0.001), graft configuration (*p *< 0.001), exposure approach (*p *= 0.001), and reconstruction of the IMA (*p *= 0.008). Of these, inflammatory AAA (*p *= 0.011), iliac PAD (*p *< 0.001), iliac aneurysm (*p *= 0.001), and graft configuration (*p *< 0.001) were independently associated with OSRT in the multivariate logistic regression model (Table 4).

**Table 4:** Univariate and multiple linear regression models for operation time.

	Univariate model			Multiple model		
	*β*	±SE	*p*-value	*β*	±SE	*p*-value
(Intercept)				**201.827**	**±17.921**	**<0.001**
Hostile abdomen	**15.463**	**±8.225**	**0.061**	12.181	±8.186	0.137
Inflammatory AAA	**38.152**	**12.142**	**0.002**	**30.909**	**±12.063**	**0.011**
AIOD	**43.620**	**8.051**	**<0.001**	**33.952**	**±8.212**	**<0.001**
Iliac aneurysm	**52.381**	**7.847**	**<0.001**	**30.881**	**±8.672**	**<0.001**
Y-graft configuration	**−56.633**	**7.019**	**<0.001**	**−40.891**	**±8.466**	**<0.001**
BMI	0.139	0.473	0.769	0.142	±0.429	0.740
Retroperitoneal approach	**37.911**	**11.334**	**0.001**	18.473	±11.260	0.102
IMA reconstruction	**33.947**	**±12.833**	**0.008**	20.104	±14.246	0.159
RAS reconstruction	21.325	27.318	0.435	25.292	±27.187	0.353

Regression parameter (*β*) ± standard error (SE).

Bold values represent the variables which were significantly different between 2 time-points.

## Discussion

While our center was early in adopting the EVAR approach, we also recognized the benefits of open surgery in certain clinical circumstances, which explains the observed stable 50:50 distribution between OSR and EVAR over the last two decades. In many international centers, the utilization of EVAR has been more pronounced, which has often been recalled in reduced OSR rates (10–12). This lower number of OSR procedures might result in less surgical training and experience, thus leading to an increased OSRT and potentially to an impaired clinical outcome.

However, a stable and even OSR workload was accompanied by a significant alteration taking place with regard to in-patient selection, case mix, and open surgical approaches. In line with international trends, patients undergoing OSR are older, and more female patients tend to undergo repair (3, 13). Comorbidities including dyslipidemia and hypertension became more prevalent, while the frequency of PAD, coronary arterial bypass graft (CABG), coronary artery disease (CAD), and smoking declined (8, 10, 14). With regard to the population assessed in the course of this study, controversial results were observed. This might be potentially explained by the improved best-medical treatment (BMT) of patients undergoing surveillance at our institution, which might have been recalled in a slight decrease of both EVAR and OSR observed between 2009 and 2017. While the institution’s strategy to preserve BMT in AAA patients remained unchanged, it might be postulated that an uprising trend with regard to the number of EVAR/OSR cases performed starting in 2017 can be attributable to an observational AAA study initiated at our institution in 2017, in the course of which more patients with AAAs were recruited. Because screening for AAAs is performed across the male population for the most part, female patients with AAAs are usually treated for late, rapidly growing aortic aneurysms (15). Therefore, a potential focus with regard to the inclusion criteria in this observational study has been put on the recruitment of female patients. Following this, a significantly higher proportion of female patients undergoing OSR during TP2 could be observed. This trend is additionally supported by evidence suggesting that a smaller proportion of female patients are eligible for EVAR since currently manufactured devices are primarily adapted for male patients (16). A number of morphological and surgical–technical significant changes attributable to OSRT could be identified in this study. For instance, the significantly more frequent utilization of more time-consuming Y-graft configuration and reconstruction of IMA was counteracted by a marked reduction in the use of retroperitoneal approaches and the fact that inflammatory AAAs, iliac aneurysms, and PAD disease became less prevalent in TP2 (Table 4). At the same time, the median proximal aortic neck length was significantly higher in patients undergoing OSR during TP2. To the best of our knowledge, such an alteration could be potentially explained by the introduction of stent grafts of the modern generation into clinical practice, which facilitated EVAR to patients presenting an aortic neck length of less than 15 mm, which was historically considered an unsuitable proximal landing zone.

The net result of these changes is an observed stable operation time and a good outcome being maintained after open AAA repair in the endovascular era (17, 18).

According to Qin et al., nowadays, only approximately 16% of all patients suffering from AAAs are treated by open repair (12). In fact, there seems to be a very strong volume–outcome relationship (17–22). The perioperative mortality rate decreases with increasing annual OSR numbers and the risk for complications (16, 23). Surgical expertise for OSR generally appears associated with reduced postoperative complication rates and improved in-hospital mortality (24). Gray et al. described a 30-day mortality rate of 6% for fewer than five open procedures per surgeon but 3% for more than 20 procedures (19). In comparison, the 30-day mortality rate in our collective was consistent between 2.4% and 3.0%.

A minimum of 30 cases/year per center seems necessary for stable long-term outcomes and the minimum number per trainee to achieve surgical expertise (19, 25). The annual number of OSRs per surgeon was fewer than three in many centers (26, 27). As a result, the proportion of OSRs in the trainee program has decreased from more than 50% to 15% in 10 years (27).

Thus, a minimum level of OSR seems reasonable and necessary to train the next generation of surgeons adequately. It could be demonstrated that the involvement of trainees has no negative impact on outcomes (6). Other methods such as simulation-based training may also be useful, and their effectiveness has recently been demonstrated (28).

With regard to this study, the proportion of vascular surgery residents in training to senior surgeons remained stable over time. Over a time span of 20 years, the experience and competence of residents in training were increasing; nevertheless, teaching cases were always supervised by the same unchanged team of five senior specialists. This, in turn, provides further evidence to suggest that OSRT remained stable without being compromised by the experience of young vascular team members instructed to perform the procedure. Despite slight fluctuations of OSR cases performed per year, it should also be noted that the ratio of senior specialists to patients undergoing open AAA repair remained well balanced at our single center. Being considered one of the major vascular surgery centers in Vienna, such a stable ratio might be explained by a concept of centralization, much attention to which has been put recently into maintaining quality medicine at a reasonable cost.

Essentially, OSR is still indicated in many cases, which is encouraged by the reported long-term survival advantage for OSR patients (29). This is also underlined by the ESVS 2019 AAA guidelines, which advocate OSR as a first-hand alternative for patients with long life expectancy (1).

Several limitations need to be acknowledged. A potential drawback of this study is its retrospective design and its single-center population. Furthermore, treatment decision between EVAR and OSR was made during a multidisciplinary discussion of each case by the same team of interventional radiologists and vascular surgeons, primarily taking into account the anatomic suitability and general health condition of a patient. However, if the case was considered suitable for both EVAR and OSR, surgeon’s and patient’s preference contributed equally to the final decision-making process, potentially presenting a limitation of the study.

## Conclusion

A balanced EVAR to OSR ratio observed during a time span of 20 years at a single center was found to be accompanied by a maintained open surgical caseload. At the same time, alterations regarding case mix, aortic aneurysm morphology, and technical aspects of the OSR approach did not lead to a reduced operation time. In addition, they resulted in satisfactory clinical outcomes following a single-center experience of open AAA repair in the endovascular era.

## Data Availability

The raw data supporting the conclusions of this article will be made available by the authors, without undue reservation.
